# Effect of Preterm Birth on Cardiac and Cardiomyocyte Growth and the Consequences of Antenatal and Postnatal Glucocorticoid Treatment

**DOI:** 10.3390/jcm10173896

**Published:** 2021-08-30

**Authors:** Amanda Vrselja, J. Jane Pillow, M. Jane Black

**Affiliations:** 1 Department of Anatomy and Developmental Biology, Monash Biomedicine Discovery Institute, Monash University, Melbourne, VIC 3800, Australia; jane.black@monash.edu; 2 School of Human Sciences, University of Western Australia, Perth, WA 6009, Australia; jane.pillow@uwa.edu.au

**Keywords:** infant, preterm, cardiac development, cardiomyocyte, developmental programming, antenatal corticosteroids, postnatal steroids, glucocorticoids

## Abstract

Preterm birth coincides with a key developmental window of cardiac growth and maturation, and thus has the potential to influence long-term cardiac function. Individuals born preterm have structural cardiac remodelling and altered cardiac growth and function by early adulthood. The evidence linking preterm birth and cardiovascular disease in later life is mounting. Advances in the perinatal care of preterm infants, such as glucocorticoid therapy, have improved survival rates, but at what cost? This review highlights the short-term and long-term impact of preterm birth on the structure and function of the heart and focuses on the impact of antenatal and postnatal glucocorticoid treatment on the immature preterm heart.

## 1. Introduction

Preterm birth is strongly associated with adverse health outcomes in the neonatal period and in later life. The long-term deleterious impacts of preterm birth only became evident over recent decades, as the first survivors of very and extremely preterm birth transitioned from infancy through childhood and adolescence to adulthood. Preterm infants are born when their organs are structurally and functionally immature. Most organ systems mature late in gestation in preparation for the many physiological changes that occur ex utero. A late gestational surge in endogenous glucocorticoids within the fetus plays a key role in this maturational process. The inverse relationship between gestational age at birth and adverse health outcomes (both short- and long-term) is largely due to the detrimental impact of preterm birth and/or factors associated with preterm birth on organogenesis.

This review focuses primarily on the structural and functional effects of preterm birth on the heart and the consequences of antenatal and postnatal glucocorticoids on the heart. Normally, cardiomyocytes (the functional units of the heart) differentiate and mature late in gestation in preparation for the major hemodynamic transition that occurs at birth, whereby there is a marked increase in left ventricular output, systemic blood pressure, and heart rate. Therefore, the heart muscle is immature when birth occurs prematurely and is ill-prepared for the postnatal haemodynamic demands; this immaturity can lead to cardiovascular impairment at birth and altered cardiac development postnatally. Cardiac development and programming for long-term cardiovascular disease may be impacted adversely by factors leading to the induction of preterm birth (such as intrauterine growth restriction and chorioamnionitis; reviewed previously by Bensley et al. [1]) or by factors associated with the antenatal care of the mother (such as treatment with antenatal corticosteroids) and the postnatal care of the infant (for example, postnatal glucocorticoid therapy).

## 2. Preterm Birth Interrupts the Normal Development and Function of the Cardiovascular System

The primitive heart is first developed and shaped in early gestation, when it is highly sensitive to spatial–temporal signalling for the induction, specification, proliferation, and differentiation of the cardiac progenitor cells [2]. As fetal development proceeds, the growth of the heart is primarily achieved via cardiomyocyte proliferation [3,4,5,6,7]. In late gestation, cardiac development progressively transitions from hyperplastic cardiomyocyte growth to hypertrophic growth, whereby the cardiomyocytes become mature and terminally differentiated in preparation for the increased haemodynamic demand at birth [6,7,8,9]. The transitional change in cardiomyocyte growth from hyperplasia (immature cardiomyocytes) to hypertrophy (mature cardiomyocytes) ultimately influences lifelong cardiomyocyte endowment and functional reserve, given the very limited replicative capacity of cardiomyocytes in postnatal life [4,10,11]. In animal models, the maturational change in cardiac growth coincides with induction of binucleation of the cardiomyocytes, which occurs when DNA synthesis and karyokinesis occur without cytokinesis [4,7,9,12,13,14]. The failure of cytokinesis to occur in fully differentiated cardiomyocytes may be due to their inability to undergo dedifferentiation, whilst maintaining their contractile function [15,16,17]. Binucleation may allow the generation of twice the genetic material, enabling cellular protein production and hypertrophy when metabolic requirements are increased in terminally differentiated cardiomyocytes [18]. In the human heart, cardiomyocytes mostly remain mononucleated as they undergo hypertrophy and commonly respond to stimuli like haemodynamic changes or stress by DNA synthesis without karyokinesis or cytokinesis [10,19]. These responses result in polyploid mononucleated cells rather than multinucleation [10,19]. However, a low cardiomyocyte turnover remains postnatally and continues up until young adulthood [10,11]. In animal models, cardiomyocytes may undergo multinucleation and polyploidy during normal postnatal development and under stressed conditions [20]. Hence, the binucleation and/or induction of polyploidy of cardiomyocytes that follows the haemodynamic transition at birth may enable cells to adapt to increased postnatal functional demands without dedifferentiation.

Premature birth abruptly interrupts the important transitional period for cardiomyocytes that normally results in cardiomyocyte differentiation in preparation for postnatal life. Consequently, preterm infants are vulnerable to myocardial structural and functional maladaptations following preterm birth due to cardiac immaturity. Preterm infants often experience difficulties in the haemodynamic transition at birth, leading to cardiopulmonary instability and complications, such as hypotension, patent ductus arteriosus, and pulmonary hypertension [21,22]. In particular, the ability of the immature myocardium to respond during the transitional period to the haemodynamic variables influencing cardiac output (including preload, contractility, afterload, and heart rate) is limited and often compensatory [23]. In preterm-born infants, the degree of prematurity is directly related to myocardial maturity, which ultimately influences myocardial contractility and the ability to cope with the haemodynamic demands [24,25,26,27].

### 2.1. Potential for Preterm Birth to Programme for Long-Term Cardiovascular Disease

It is clear that early life events impact organ development and function deleteriously, and thus programme infants for increased vulnerability to developing adulthood disease [28,29]. Postnatal organ injury and adaptive remodelling of the immature organ systems can lead to lifelong adverse impacts on organ function, given that preterm infants are born with structurally and functionally immature organ systems. For example, the association between preterm birth and increased blood pressure is observed from as early as 2.5 years of age [30]. An inverse relationship between gestational age at birth and levels of blood pressure has been evident in population-based studies in childhood [31,32], and in adolescence and adulthood [33,34,35]. The increase in blood pressure in those born preterm likely renders vulnerability to the development of cardiovascular disease, given that hypertension is a major risk factor for cardiovascular disease and is often associated with left ventricular hypertrophy [36,37,38,39]. Another contributing factor influencing the cardiovascular phenotype following preterm birth is whether the infant was born of appropriate weight for gestational age or small for gestational age, a proxy for fetal growth [40]. A well-described example of developmental programming is the link between low birth weight and the risk of cardiovascular disease, in particular coronary heart disease [41,42,43,44,45]. Low birthweight can result from intrauterine growth restriction and/or preterm birth. Alarmingly, mounting evidence links preterm birth (with poor or normal fetal growth, assessed by birth weight) to the early-life origins of cardiovascular disease, including increased risk of early heart failure and ischaemic heart disease in later life [46,47].

### 2.2. Maladaptive Structural Remodelling and Functioning of the Heart Following Preterm Birth

Examination of the impact of preterm birth on the heart in animal studies requires the use of an animal model that closely reflects the development of the human heart. For example, sheep are a highly relevant experimental model for this purpose, due to similarities in the late gestational timing of cardiomyocyte growth and maturation between sheep and human hearts [4,6]. An important study from Bensley et al. [48] showed that preterm birth induced marked changes in the cardiac structure of moderately preterm lambs (born at 133 days of gestation; term is at 147 days of gestation), including increased myocardial collagen deposition and accelerated cardiomyocyte growth and maturation at 9 weeks post-term equivalent age [48]. Cardiomyocyte number was not different between the hearts of preterm and term-born sheep. However, cardiomyocyte maturation was perturbed in response to preterm birth, including increased ploidy of mononucleated cardiomyocytes and cardiomyocyte hypertrophy [48]. These important findings show that preterm birth leads to maladaptive cardiac remodelling in the early postnatal period [48] and are supported by data from other animal models highlighting the functional and structural changes of the immature preterm heart [49,50,51]. Extensive investigations over the past decade in animals and humans have demonstrated the impact of being born early on the heart.

Cardiovascular imaging studies have highlighted the gross structural and functional changes of the heart in infants and adults born preterm. Pivotal studies conducted in human subjects by Lewandowski and colleagues [52,53] investigated the long-term impact of preterm birth on the heart structure and function using cardiac magnetic resonance imaging. Their novel findings highlighted that young adults born preterm subsequently exhibit both a unique cardiac geometry and impaired cardiac function in early adulthood compared to those born at term. Preterm-born young adults exhibited shorter ventricles and increased ventricular wall thickness with a decrease in internal luminal diameter in both the right and left ventricles [52,53]. There was also apical displacement away from the right ventricle in the hearts of the preterm-born adults when compared to those born at term. The structural changes to the heart were coupled with significant impairments of systolic and diastolic function, including reductions in cardiac output, stroke volume, end-systolic volume, longitudinal peak systolic strain, peak systolic and diastolic strain rate, and altered rotational movement [52,53]. The changes in right and left ventricular mass were inversely proportional to gestational age at birth: right ventricular mass increased by 2.74% and left ventricular mass increased by 1.47% for every week of reduction in gestation [52,53]. Similar findings of altered cardiac structure and function were highlighted by other imaging studies of adults born prematurely [54,55]. The reported increases in left ventricular mass in subjects born preterm [52,55] is concerning given that left ventricular hypertrophy is a major independent predictor for cardiovascular disease [56,57,58,59].

In contrast, some experimental studies have identified lower cardiac mass in the hearts of adolescents and young adults born preterm [60,61]. Late-adolescents who were born extremely preterm showed evidence (on M-mode echocardiography) of minor cardiovascular effects due to prematurity, including increased blood pressure and reduced left ventricular mass but preserved left ventricular function [60,61]. The differences in ventricular mass findings between studies are currently unknown but are likely due to differences in methodology between studies. Given many of the clinical studies have mixed cohorts of preterm-born individuals, it is important to consider that some of the cardiovascular features attributed to preterm birth may be related to the compromised growth in utero (small for gestational age vs. appropriate weight for gestational age), as well as the degree of prematurity, perinatal care, and/or the levels of blood pressure in the subjects studied.

The increased blood pressure and increased cardiac mass in young adults born preterm likely arise consequent to events during the perinatal period: cardiac magnetic resonance imaging studies have shown clear evidence of cardiac changes first emerging early in life [62,63]. Preterm infants in the early postnatal period (soon after delivery to 3 months of age) have a disproportionate increase in ventricular mass to body and cardiac size [62,64], and increased intraventricular septal thickness [65] and altered right ventricular structure and function [66] are evident by early childhood. Interestingly, increased left ventricular myocardial wall thickness in preterm neonates was independently associated with the degree of prematurity, antenatal glucocorticoid administration, and requirement for postnatal respiratory support [63]. Whether these changes in cardiac mass of preterm-born individuals are mediated by hemodynamic changes and the effects on blood pressure or antenatal experiences and exposure to neonatal critical care are yet to be elucidated.

The cardiac changes associated with prematurity are of major clinical concern given the vulnerability of the hearts of preterm-born individuals to exercise and an increased incidence of heart failure from childhood through to adulthood [46,47,55,67,68]. Notably, young adults born preterm respond with both impaired left ventricular (reduced ejection fraction and submaximal cardiac output response [55]) and right ventricular function (reduced capacity to augment cardiac index and stroke volume from rest [67]) when challenged with the physiological stress of physical exercise. Recently, Huckstep et al. [68] reported that normotensive young adults born preterm exhibited reduced aerobic exercise capacity and slowed heart rate recovery compared to healthy young adults born at term; the impairment in aerobic capacity and heart rate recovery was related to the level of impairment with left ventricular systolic function and exercise intensity. The impaired functional responses of the heart to the challenges of physical exercise in young adults born preterm is suggestive of reduced myocardial functional reserve [55,67,68]. A perinatal autopsy study conducted by Bensley et al. [69] demonstrated that preterm-born infants have a marked reduction in the proportion of proliferative cardiomyocytes relative to age-matched stillborn infant controls. The reduction in proliferative cardiomyocytes suggests impaired cardiomyocyte endowment in response to preterm birth. Indeed, a reduced functional reserve in the hearts of preterm-born individuals may explain the relationship between preterm birth and the increased risk of early heart failure and ischemic heart disease [46,47]. A recent meta-analysis investigated the cardiac differences between term- and preterm-born individuals and demonstrated that being born premature is associated with changes in cardiac structure and function from birth to young adulthood [70]. The unique cardiac phenotype of indivduals born preterm may be more vulnerable to secondary insults, which may contribute to their risk of cardiovascular disease [70].

### 2.3. Improved Survival of the Most Vulnerable Preterm Infants—Implications for Cardiac Health

Recent advances in neonatal medicine have markedly reduced the incidence of neonatal mortality and morbidity of the most vulnerable preterm populations. The improvement in the survival of infants born preterm is largely attributed to the now routine administration of antenatal steroids to mothers at risk of delivering prematurely. In 1972, the first randomised trial of antenatal corticosteroid treatment for the prevention of respiratory distress syndrome was conducted by Liggins and Howie [71]. Now, women at risk of preterm birth routinely receive corticosteroids to accelerate lung maturation of the fetus to enhance lung function, gas exchange, and survival of the infant if preterm birth ensues [72]. The synthetic glucocorticoids, betamethasone and dexamethasone, are the preferred corticosteroids for antenatal therapy as they readily cross the placenta [73,74].

Postnatal care plays a crucial role in assisting the premature transition from fetal to postnatal life and improves the survival of the most vulnerable preterm infants. Some infants still develop respiratory distress syndrome, and will also receive exogenous surfactant to reduce the severity of respiratory disease and also to reduce neonatal mortality [75,76]. Although surfactant improves pulmonary compliance, extremely preterm neonates with pulmonary immaturity commonly experience significant respiratory distress. The compromised respiratory function and consequent treatment, including mechanical ventilation and use of hyperoxic gas mixtures, increase susceptibility to chronic pulmonary conditions, such as bronchopulmonary dysplasia and a dependency on ventilation. In such a clinical setting, administration of glucocorticoids postnatally may facilitate weaning from respiratory support and prevent further respiratory failure [77]. Of interest, infants treated with postnatal glucocorticoids often develop cardiac hypertrophy [78,79,80,81]. Whether this glucocorticoid-induced cardiac hypertrophy results in permanent structural modifications in the preterm heart is not yet known.

There is widespread use of glucocorticoid treatment (both antenatally and postnatally) for improvement of preterm birth outcomes. However, the treatment varies globally in clinical practice, including the type of glucocorticoid used (betamethasone, dexamethasone, or hydrocortisone), dose and duration of glucocorticoid course, and the route of administration. To date, there is no consensus on best practice for postnatal glucocorticoid treatment.

## 3. Corticosteroids

Corticosteroids are an essential class of steroid hormones that regulate a variety of physiological processes, such as energy metabolism, inflammation, and organ maturation. Corticosteroids are produced by the adrenal cortex, under the control of the hypothalamic-pituitary–adrenal (HPA) axis. Cortisol is the major glucocorticoid end-product of the HPA axis. Glucocorticoids mediate their effects on almost every tissue in the body via binding to the ubiquitously expressed glucocorticoid receptor [82,83].

Cortisol, an endogenous glucocorticoid, is vital for fetal organ maturation in preparation for postnatal life and is tightly regulated by the enzyme 11β-hydroxysteroid dehydrogenase type 2 (11β-HSD2) during gestation. 11β-HSD2 is highly expressed in the placenta, where it converts cortisol into its inactive form (cortisone) thereby restricting fetal exposure to maternal cortisol [84]. The placental activity of 11β-HSD2 declines towards late gestation, coinciding with an increase in fetal cortisol levels just before term [73,84]. The late gestational surge in cortisol encourages maturation of fetal tissues and organs to prepare for the transition from intrauterine to extrauterine life [85,86]. In sheep, there is an exponential rise in fetal cortisol levels around 20 days before term birth (approximately 130 days of gestation, term is ~150 days of gestation) [87]. Interestingly, animal studies have shown that the rise in cortisol coincides with the late gestational changes in cardiomyocyte growth kinetics, the gradual decline in cell cycle activity and the increase in cell size and binucleation [6,7]. Importantly, glucocorticoids exert direct effects on the heart, which are mediated in cardiomyocytes by the glucocorticoid receptor and mineralocorticoid receptor [82].

### 3.1. Exogenous Glucocorticoids as an Antenatal Treatment to Women at Risk of Preterm Birth

The late gestational rise in cortisol is mimicked clinically with the antenatal administration of the synthetic glucocorticoids, mainly betamethasone or dexamethasone [88]. Both betamethasone and dexamethasone are poor substrates for 11β-HSD2 and, therefore, readily cross the placenta. They are both devoid of mineralocorticoid activity and bind with high affinity to the glucocorticoid receptor, similarly to the endogenous ligand (cortisol). However, the potency of betamethasone and dexamethasone is 25–50 times greater than cortisol [74]. Betamethasone treatment is available in two forms and for maximal drug efficacy is generally administered as a combination of both betamethasone sodium phosphate (short biological half-life of 36–72 h) and betamethasone acetate (slow tissue absorption and relatively longer biological half-life) [74,88]. Dexamethasone treatment is generally given as dexamethasone sodium phosphate, which has a short half-life of 36–72 h [74]. Currently, the recommended clinical course is two doses of 12 mg of betamethasone administered intramuscularly at 24 h apart or four doses of 6 mg of dexamethasone administered intramuscularly at 12 h apart [89]. These recommended doses are comparable to physiological stress levels of cortisol in the fetus and occupy a high number of glucocorticoid receptors in fetal tissues [89,90]. Synthetic glucocorticoids are used routinely in clinical practice as an antenatal prophylactic treatment for preterm birth because they can facilitate fetal lung maturation at a much earlier timepoint in gestation than occurs naturally. Although antenatal corticosteroid therapy has proven to be an effective treatment, there is conflicting information on the effect of excess glucocorticoid exposure in utero on cardiovascular outcomes.

### 3.2. Antenatal Corticosteroids Influence Maturation of the Immature Heart

Glucocorticoid signalling during fetal development is critical for structural, functional, and biochemical maturation of cardiomyocytes [91,92]. Animal studies in models mimicking clinical treatment or chronic maternal stress [93,94,95,96,97,98,99] indicate that there are timing, dose-dependent, and experimental model differences in the response to antenatal corticosteroid exposure in relation to fetal cardiac growth and maturation outcomes. These findings are sometimes conflicting. Table 1 provides a summary of animal studies investigating the antenatal effects of glucocorticoids on the heart. For example, dexamethasone treatment in early gestation had no effect on fetal body and heart weight in sheep [94] whereas betamethasone treatment in late gestation reduced both fetal body and heart weight. Hence, glucocorticoid treatment late in gestation can directly influence the growth of the fetus [94]. The reduction in heart size directly related to the overall impact on body size [94]. In contrast, other late-gestation ovine studies have reported induction of cardiac hypertrophy following exposure to antenatal glucocorticoids (including hydrocortisone; a physiological replacement for cortisol) with increases in heart weight and heart weight to body weight ratio [95,96,97]. The increased heart size is most likely due to direct influences of the antenatal glucocorticoids on cardiomyocyte proliferation and/or hypertrophy. Although preterm piglets exposed to maternal betamethasone treatment do not exhibit changes in heart weight or relative heart weight to body weight, they do show altered cardiomyocyte maturation, including decreased proliferation and increased apoptosis of cardiomyocytes as well as an increased proportion of binucleated left ventricular cardiomyocytes [98,100].

Late gestational cortisol infusion in sheep increases blood pressure, fetal heart to body weight ratio, left ventricular cardiomyocyte size, and cardiac angiotensinogen mRNA: these outcomes are consistent with accelerated cardiovascular maturation mediated by glucocorticoid exposure [97]. The hypertrophic growth in left ventricular cardiomyocytes is potentially a physiological response to the increase in blood pressure and cardiac angiotensinogen mRNA levels [97]. Reini and colleagues [102] reported a similar increased fetal heart to body weight ratio and increased ventricular wall thickness after cortisol infusion in late gestation. However, the morphological cardiac changes were not due to changes in blood pressure or cardiac fibrosis, suggesting overexposure to cortisol in fetal sheep directly influenced cardiomyocyte growth by acting on the cardiac mineralocorticoid and glucocorticoid receptors [102].

In a model of maternal stress, fetal sheep chronically exposed to maternally administered cortisol during late gestation exhibited relatively larger heart to body weight ratios, increased ventricular wall thickness, and increased mean arterial blood pressure [95]. Interestingly, augmented cardiac growth was attributed to an accelerated hyperplastic growth of cardiomyocytes, evidenced by an increased proportion of cardiomyocytes stained positive for Ki-67 and no change in cardiomyocyte size [95]. Increased maternal cortisol levels during late gestation by cortisol infusion also stimulated both proliferation and apoptosis in the fetal sheep hearts [103]. Clearly, chronic exposure to maternal cortisol triggers cardiac remodelling and altered growth trajectory during the critical maturational transition period for cardiomyocytes.

In a fetal growth-restricted model (risk factor for preterm birth), betamethasone-exposed sheep fetuses showed increased relative heart weight and exhibited enhanced left ventricular responsiveness to β-adrenoceptor stimulation, which may lead to cardiac dysfunction [105]. Cardiac function is highly dependent on the electrical activity of cardiomyocytes and their ability to propagate signals, which is governed by ion channels such as the voltage-gated sodium channels [104]. Cortisol has a role in regulating sodium channels and their subunits during fetal sheep development [104]. Hence, overexposure of fetal sheep to cortisol during development leads to precocious increases in the expression of sodium channels and could potentially impair cardiac function [104]. Reduced cardiac functional reserve alongside left ventricular hypertrophy was observed in adult sheep that were exposed to dexamethasone early in gestation [101].

Prenatal exposure of rat pups to dexamethasone results in relatively larger hearts and increased proliferative index, suggesting delayed cardiac maturation [93]. In adulthood, rats that were antenatally exposed to dexamethasone have enhanced expression of calreticulin, a Ca++ binding protein that interacts with the glucocorticoid receptor; the overexpression of calreticulin is linked to impaired cardiac function [106]. Corticosterone treatment of fetal mice cardiomyocytes in vitro acts mainly through the glucocorticoid receptor to promote cardiomyocyte contractility and also cytoskeletal structure, such as myofibril assembly and sarcomeric organisation [91]. A conditional glucocorticoid receptor knockout results in mice with smaller hearts, poorly aligned cardiomyocytes with disorganised myofibrils, and a failure to induce genes involved in cardiac function, such as contractility, calcium handling, and metabolism [92]. In rodents, cardiomyocyte growth continues via proliferation in the early postnatal period before switching to predominantly hypertrophic growth and, therefore, there is an early postnatal window of opportunity for cardiac changes in response to postnatal life [12,13,14,93]. Hence, the effects of antenatal corticosteroids on the immature heart may be different in rodents compared to large animal models, such as baboons, sheep and pigs, which have a similar cardiac maturational profile to humans.

There have been few investigations into the long-term cardiovascular side effects of antenatal glucocorticoid exposure. Insights from magnetic resonance imaging in adult male baboons who were exposed to antenatal synthetic glucocorticoids highlighted an increase of 150% in pericardial fat thickness (a marker of metabolic cardiac health [107,108]) with unchanged body weight compared to controls [99]. Additional investigations identified metabolic biomarkers of pericardial fat and increased body weight and serum lipids in adult male baboons exposed to antenatal betamethasone [109]. Metabolic dysregulation and obesity are established risk factors for cardiovascular disease [110].

In addition to the direct effects on the growth and maturation of the heart, antenatal glucocorticoid exposure impacts cardiovascular physiology. The administration of betamethasone to pregnant baboons, in doses equivalent to those administered to pregnant women at risk of preterm delivery, results in significant cardiovascular changes in the fetus, and in particular, increased blood pressure [111]. Elevated blood pressure consequent to antenatal glucocorticoid treatment has been observed in other nonhuman primate studies of the premature neonate and in juvenile offspring [112,113,114]. These findings are supported by glucocorticoid increased fetal blood pressure and altered basal cardiovascular function after fetal exposure to exogenous glucocorticoids in sheep [96,115,116]. Changes to systemic and pulmonary pressures and resistance are known stimuli for cardiomyocyte growth and maturation in fetal lamb studies [6,117,118,119]. From a clinical perspective, modulators of cardiac growth and endowment, such as antenatal exposure to exogenous glucocorticoids coupled with changes in blood pressure, may have long-term consequences on the preterm-born individual.

There is limited clinical literature in infants relating to the postnatal cardiac effects of antenatal corticosteroid exposure. A prospective study [120] examining routine administration of maternal betamethasone did not identify altered cardiac dimensions in preterm neonates. In contrast, repeated courses of antenatal glucocorticoids led to transient hypertrophic cardiomyopathy in three infants born preterm [121]. Collectively, the findings from experimental and clinical studies highlight the complexity of interpreting the effects of antenatal corticosteroid exposure on cardiac outcomes with findings between studies confounded by differences in timing exposure, dose, glucocorticoid administered, and species studied. However, antenatal glucocorticoid administration clearly influences cardiac maturation.

### 3.3. Use of Steroids in Postnatal Treatment for Preterm Infants

In contrast to the complex story regarding the effect of antenatal glucocorticoids on heart development, strong clinical evidence has highlighted the common development of transient cardiac hypertrophy in preterm neonates administered postnatal glucocorticoids. Postnatal steroid therapy accelerates weaning from mechanical ventilation, improves respiratory outcomes of preterm infants with severe bronchopulmonary dysplasia, and prevents respiratory failure [77]. These benefits of postnatal glucocorticoid therapy are present if they are administered early (<8 days) or later in the postnatal period [122,123]. The efficacy of postnatal glucocorticoids for the treatment of severe bronchopulmonary dysplasia is, in part, related to their strong anti-inflammatory properties, which downregulate persistent pulmonary inflammation. Nonetheless, administration of postnatal glucocorticoids remains a highly contentious neonatal practice [124], and hence, their use has been tempered over time. High-dose dexamethasone was used initially and considered promising as there was a rapid improvement of respiratory status in treated preterm newborns. However, the subsequent side effects were alarming and included poor growth, hypertension, hypertrophic cardiomyopathy, infection, and neurodevelopmental delay [122,123]. As a result, high-dose postnatal dexamethasone therapy was withdrawn from use in the neonatal intensive care unit. However, given the beneficial anti-inflammatory effects of steroids in the lungs of the preterm newborn, lower doses of postnatal steroids are now used in the neonatal intensive care unit because they confer a therapeutic benefit without a number of negative side effects. In the first of the clinical studies investigating low-dose postnatal steroid therapy in preterm neonates, no definitive conclusions could be drawn due to termination of the study because of low patient recruitment [125,126]. More recently, postnatal steroid therapy at a low-dose was re-introduced into the clinic for the care of preterm neonates with encouraging outcomes from clinical trials, including no association of low-dose steroid treatment with adverse neonatal events or neurodevelopment outcomes at 2 years of age [127,128].

### 3.4. Transient Hypertrophic Cardiomyopathy in Preterm Infants

One adverse consequence following dexamethasone treatment in preterm infants is the induction of hypertrophic cardiomyopathy, characterised as hypertrophy of the left ventricle and interventricular septum. The degree and rate of increases in left ventricular and septal wall thickness vary case by case, in response to the postnatal steroid therapy [78,79,80,81]. Myocardial hypertrophy is a common side effect following postnatal dexamethasone treatment and has been reported in premature infants administered a 42-day course [80,129], 2–4-week courses [78,130], 7-day course [81,131], and a very short 3-day pulse course [132] of dexamethasone therapy. Some infants with dexamethasone-induced myocardial hypertrophy further develop left ventricular outflow tract obstruction [78,79,133]. The onset of hypertrophy occurs very quickly after dexamethasone treatment commences but resolves quite rapidly once the infant is weaned from dexamethasone [78,81,130]. It remains unclear whether dexamethasone-induced myocardial hypertrophy leads to persistent alterations in cardiomyocyte growth and cardiac structure.

### 3.5. Dexamethasone-Induced Cardiac Changes in Experimental Models

Postnatal dexamethasone treatment in the early postnatal period induces transient cardiac hypertrophy in rats [134,135,136,137]. Table 2 provides a summary of the limited preclinical studies investigating the effects of postnatal glucocorticoid administration on the development of the neonatal heart. High-dose dexamethasone treatment in rat pups (equivalent to a 21-day tapering dose in humans) results in perturbed cardiac growth in the neonatal period, including reduced heart and left ventricular weight, and reduced left ventricular wall and lumen volume [138,139,140]. In addition to altered cardiac growth, dexamethasone therapy reduces left ventricular systolic function in rats and increases wall stress [141]. At the cellular level, early postnatal dexamethasone treatment induces cardiomyocyte hypertrophy in rat pups but this effect normalises within a short period following cessation of dexamethasone treatment [140], although some studies have shown that the cardiomyocyte hypertrophy reappears in adulthood [139,142].

High-dose dexamethasone treatment in newborn rats (postnatal day 1 to day 3) perturbs normal cardiomyocyte development by stimulating accelerated maturation and inhibiting proliferation [143]. Neonatal cardiomyocytes from dexamethasone-treated pups exhibited marked changes in cardiomyocyte growth throughout the early postnatal period when compared to rat pups that were not treated with dexamethasone: at postnatal day 4, increased binucleation and decreased proliferation; at postnatal day 7, decreased proliferation persisted; and at postnatal day 14, total cardiomyocyte number was reduced but heart to body weight ratio was increased [143]. Dexamethasone treatment with a glucocorticoid receptor antagonist (RU486) abrogated the effects of dexamethasone [143]. Further experiments highlighted novel DNA methylation in cardiomyocytes of neonatal rats treated with dexamethasone, which could be due to the glucocorticoid receptor-response mediated by dexamethasone [143]. Follow up studies demonstrated that dexamethasone acts through the glucocorticoid receptors to mediate inhibition of proliferation and increased binucleation of cultured neonatal rat cardiomyocytes [147].

In vitro studies with neonatal rat cardiomyocytes further implicate glucocorticoids in the development of cardiac hypertrophy [83,148,149]. Cultured neonatal rat cardiomyocytes treated with dexamethasone have increased cell size and upregulation of markers for cardiomyocyte hypertrophy, including atrial natriuretic factor, β-myosin heavy chain and skeletal muscle α-actin [83]. Interestingly, when cultured cardiomyocytes treated with dexamethasone were challenged with either serum deprivation or TNF-α exposure (which normally triggers apoptotic events), there was inhibition of apoptosis [83]. Clearly, glucocorticoid signalling influences cardiomyocyte growth, survival, and function.

### 3.6. Neonatal Dexamethasone Effects May Persist or Reappear in Adult Animal Models

The effects of early postnatal dexamethasone exposure may persist or reappear later in life in rats. The reduction in heart and body weight persists to at least eight weeks of age but normalises by 50 weeks of age [144]. In contrast, another study reported decreased body and heart weights, increased left ventricular mass, and impaired cardiac function at approximately 15 weeks of age in rats exposed to neonatal dexamethasone [145]. Although there are limited reports on the changes during adulthood after postnatal dexamethasone treatment [144,145], the reduction in heart and body weight is evident in elderly rats (80 weeks of age) [144]. In addition to reduced heart and body weight, cardiac dysfunction was observed at four weeks of age [141] and normalised by eight weeks of age, but then reappeared by 50 weeks of age and became more pronounced by 80 weeks of age [144]. De Vries and colleagues [142] also observed cardiac hypertrophy at 45 weeks of age in rats treated with dexamethasone during the early neonatal period, which was accompanied with the development of left ventricular wall thickening and increased myocardial interstitial fibrosis at 15 months of age [146]. Collectively, these rat studies highlight that the transient cardiac changes due to postnatal dexamethasone treatment in the early neonatal period may persist or reappear along with other structural cardiac changes in adulthood and be further amplified with age.

Together, these findings suggest that early postnatal dexamethasone treatment has negative effects on cardiac growth and function during the important period of cardiac maturation, and these negative effects may persist into adulthood. Rodents are a useful model for studying the immature heart because the switch in cardiomyocyte growth from hyperplasia to hypertrophy occurs in the early postnatal period [12,13]. However, the effects of postnatal steroid therapies on the heart have only been investigated in rodents after exposure to high glucocorticoid doses, which are no longer used in clinical practice, and in in vitro studies. Furthermore, it is difficult to draw relevant clinical conclusions from rodent studies given that the maturational profile of cardiomyocytes in early postnatal life is very different to those in humans [10,11,12,13,14,16]. The mechanism behind dexamethasone-induced hypertrophic cardiomyopathy and lifelong effects of dexamethasone exposure on the heart remains unclear and is an important area of research.

## 4. Future Directions

Although it is now established that preterm birth programmes a unique cardiac phenotype, the antenatal and postnatal factors also play a major role during key developmental windows of cardiac development. Future studies are required to address the impact of the administration of antenatal glucocorticoids on the heart, particularly as it is routine clinical practice to administer antenatal glucocorticoids to women at risk of preterm birth. Studies involving a preclinical large animal model (such as the lamb) permit the investigation of the effects of maternal glucocorticoid administration at varying doses at a number of gestational timepoints on cardiac development, whereby the hearts of fetuses are examined without the confounding influence of preterm delivery. Furthermore, the long-term cardiac outcomes in response to low-dose postnatal steroid therapy have not been elucidated; only high-dose steroid therapy has been examined in rats. It is concerning that our understanding to date, in relation to the impact of postnatal steroids on the heart, is limited, because there are now many people in adulthood who experienced low- and/or high-dose exposure to postnatal steroid therapy as a preterm infant. Future preclinical studies, including cardiovascular imaging, functional, morphological, and molecular studies, are required to better understand cardiac physiological adaptations and maladaptations related to preterm birth and the consequences of antenatal and postnatal glucocorticoid therapy.

## 5. Conclusions

In conclusion, preterm birth is a catalyst for a unique cardiac phenotype. Preterm infants are vulnerable to myocardial structural and functional maladaptations following premature birth due to cardiac immaturity. Furthermore, the myriad of antenatal and postnatal exposures faced by preterm infants may influence cardiac structure and function. Glucocorticoids are essential for the survival of very and extremely preterm infants; however, our current understanding of synthetic glucocorticoids in the programming of the fetal and neonatal heart is controversial. There are now many people in adulthood who experienced antenatal corticosteroid exposure and/or exposure to postnatal steroid therapy as a preterm infant. Given that cardiovascular disease takes many decades to manifest, it is highly likely that those individuals born very and extremely preterm will exhibit accelerated and exacerbated cardiovascular disease. Ultimately, the cardiovascular health of individuals born preterm needs to be monitored throughout life, as the life-long cardiovascular consequences of glucocorticoid exposure are of major clinical importance.

## Figures and Tables

**Table 1:** Effects of antenatal glucocorticoid administration on the heart.

Model	GC	Timing of Exposure	Route & Duration of Exposure	Age of Analysis	Cardiac Effects	Cardiomyocyte Characteristics	Ref.
Baboon	BETA	Mid–Late (0.6, 0.64, & 0.68 gestation)	IM; 3 courses of 2 doses	Adult (10 y)	↑ mid-ventricular pericardial fat thickness; ↔ body weight	N/A	[99]
Sheep	DEX	Early (27 d GA)	IV; 2 d	Adult (7 y)	↑ LV wall mass; ↑ LV type I collagen content; ↓ cardiac functional reserve; ↑ blood pressure	N/A	[101]
Sheep	DEX	Early (40–42 d GA)	IM; 4 doses over 48 h	Fetal (range: 49–142 d GA), Pubescent (7 m)	↔ heart weight; ↔ heart: body weight ratio	N/A	[94]
Sheep	BETA	Late (104–118 d GA)	IM; 1 every 7 d (up to 3 doses)	Fetal (range: 109–145 d GA), Pubescent (6 or 12 w)	↓ heart weight (at 122 & 132 d GA) when exposed to three doses (at 104, 111, & 118 d GA)	N/A	[94]
Sheep	HC	Late (118–123 d GA)	IV; 10 d	Fetal (129–132 d GA)	↑ heart weight; ↑ LV & RV wall thickness; ↔ interstitial collagen content; ↔ blood pressure	N/A	[102]
Sheep	HC	Late (~126–127 d GA)	IV; 2–3 d	Fetal (128–130 d GA)	↔ heart, LV, RV & IVS weight; ↑ heart: body weight ratio; ↑ LV: body weight ratio; ↑ RV: body weight ratio; ↑ mean arterial pressure; ↑ systolic & diastolic blood pressure	↔ nuclearity; ↔ number; ↑ LV mononucleated volume; ↓ LV multinucleated volume; ↔ RV volume;	[97]
Sheep	HC	Late (118–123 d GA)	IV; 10 d	Fetal (129–132 d GA)	N/A	↑ KI67 positive nuclei; ↑ apoptosis	[103]
Sheep	HC	Late (~126–128 d GA)	IC; 7 d. IV; 2 d	Fetal (134 d GA)	↑ heart weight; ↑ heart: body weight ratio	↑ KI67 positive nuclei; ↔ binucleation; ↔ length; ↔ width	[95]
Sheep	HC	Late (119 d GA)	IV; 10 d	Fetal (128 d GA)	↔ heart weight; ↑ heart: body weight ratio; ↑ LV & RV wall thickness; ↑ mean arterial pressure	N/A	[96]
Sheep	HC	Late (~121–124 d GA)	IV; 5 d	Fetal (125–130 d GA)	↑ expression of sodium channels & subunits	N/A	[104]
Sheep	BETA	Late (~115–120 d GA)	IM; 2 doses over 48 h	Fetal (117–123 d GA)	↑ heart: body weight ratio; ↔ basal contractile LV or RV function; ↑ LV responsiveness to β-adrenoceptor activation	N/A	[105]
Pig	BETA	Late (91–92 d GA)	IM; 2 doses over 48 h	Fetal (91–92 d GA)	↔ heart weight; ↔ heart: body weight ratio; ↑ atria weight; ↑ LV free wall weight (females only)	↓ KI67 positive nuclei; ↑ apoptosis; ↑ LV binucleation; ↔ volume	[98,100]
Rat	DEX	Late (17 d PC)	SC; 4–5 d	Neonatal (1 d)	↑ heart: body weight ratio; ↓ myocardial extracellular matrix	↑ proliferative index	[93]
Rat	DEX	Late (15–21 d PC)	SC	Fetal (21 d PC) to Adulthood (up to 24 w)	↓ calreticulin protein expression (fetal); ↑ calsequestrin protein expression (fetal); ↑ calreticulin protein expression (adulthood)	N/A	[106]
Mice	GR KO	Fetal	N/A	Fetal (E 17.5 d)	↓ heart size; ↓ ventricular volume; impaired cardiac function; short, disorganized myofibrils; myofibrils fail to align in the myocardium	N/A	[92]
Neonatal mouse CM	CORT	Treated for 24 h	In vitro	Fetal	N/A	↑ contractility; ↑ Z-disc assembly; ↑ sarcomere length; ↑ appearance of mature myofibrils; ↑ mitochondrial activity; ↑ contraction & relaxation events	[91]

BETA, betamethasone; CM, cardiomyocyte; CORT, corticosterone; d, day; d GA, days of gestational age; d PC, days post-conception; DEX, dexamethasone; E, embryonic; GC, glucocorticoid; GR KO, glucocorticoid receptor knockout; h, hours; HC, hydrocortisone; IC, intracoronary; IM, intramuscular; IV, intravenous; IVS, interventricular septum; LV, left ventricle; m, months; N/A, not applicable; RV, right ventricle; SC, subcutaneous; w, weeks; y, years; ↑, increase; ↓, decrease; ↔, no change.

**Table 2:** Effects of postnatal glucocorticoid administration on the heart.

Model	GC	Timing of Exposure	Route of Exposure	Age of Analysis	Cardiac Effects	Cardiomyocyte Characteristics	Ref.
Rat	DEX	1–3 d	IP injection	Neonatal (2, 4, 7 & 21 d)	↓ heart weight (21 d)	↓ mitotic index and proliferation (2 & 4 d); ↔ apoptosis (7 d); ↓ number per unit of myocardial area (7 d)	[140]
Rat	DEX	1–3 d	IP injection	Neonatal (2, 7 & 14 d)	↓ heart weight; ↑ heart: weight ratio (7 d), suggestive of transient cardiac hypertrophy	↑ cell size (7 d); ↑ expression of genes involved in fatty acid utilization (7 d)	[135]
Rat	DEX	1–3 d	IP injection	Neonatal (4, 7 & 14 d)	↑ heart weight (7 & 14 d)	↓ proliferation (4 & 7 d); ↑ binucleation (4 d); ↓ number (14 d)	[143]
Rat	DEX	1–5, or –7d	IP injection	Neonatal (5 & 7 d)	↑ heart: weight ratio, suggestive of transient cardiac hypertrophy; ↑ protein: DNA ratio	N/A	[136]
Rat	DEX	1–3, –5, –7 or –9 d	SC injection	Neonatal (1–9 d)	↑ heart: weight ratio, suggestive of transient cardiac hypertrophy	↑ expression of α-MHC; ↓ expression of β-MHC	[137]
Rat	DEX	1–5 d	SC injection	Neonatal (7 & 21 d)	↓ heart weight (7 d); ↑ heart: weight ratio, suggestive of transient cardiac hypertrophy (7 d); ↑ LV free wall-to-chamber ratio (7 d); ↔ heart: weight ratio (21 d)	N/A	[134]
Rat	DEX	1–3 d	IP injection	Neonatal (21 & 22 d)	↓ heart weight; ↓ LV wall volume; ↑ LV lumen	N/A	[138]
Rat	DEX	1–3 d	IP injection	Neonatal (7 d), Adolescent (8 w) & Adulthood (45 w)	↓ heart weight (7 d and 45 w)	↑ protein: DNA ratio (45 w); ↑ cell volume (45 w) suggestive of cardiomyocyte hypertrophy	[142]
Rat	DEX	1–3 d	IP injection	Pre-pubertal (4 w)	↓ ventricular weight; ↓ ventricular volume; ↓ contractility; ↑ wall stress	N/A	[141]
Rat	DEX	1–3 d	IP injection	Pre-pubertal (4 w), Adolescent (8 w) & Adulthood (50 w)	↓ ventricular weight (4 & 8 w); ↑ myocardial collagen (50 w); ↑ presence of macrophages (50 w)	↑ cell volume (50 w)	[139]
Rat	DEX	1–3 d	IP injection	Adolescent (8 w) & Adulthood (50 & 80 w)	↓ ventricular weight (8 & 80 w); systolic dysfunction (50 & 80 w)	N/A	[144]
Rat	DEX	1–3 d	IP injection	Adulthood (~15 w)	↓ heart weight; ↑ LV mass; cardiac dysfunction	N/A	[145]
Rat	DEX	1–3 d	IP injection	Adulthood (15 m)	Hypertrophic heart; ↑ LV wall thickness; ↑ interstitial fibrosis	↑ cell length; ↑ cell diameter	[146]
Rat neonatal CM	DEX	Treated for 48 h	In vitro	Neonatal	N/A	↓ proliferation; ↑ binucleation	[147]
Rat neonatal CM	DEX	Treated for 72 h	In vitro	Neonatal	N/A	↑ cell size; ↑expression of cardiac hypertrophic markers	[83]
Rat neonatal CM	DEX	Treated for 48 h	In vitro	Neonatal	N/A	↑ cell size; ↑ L-type Ca^++^ current density	[148]
Rat neonatal CM	CORT	Treated for 48 h	In vitro	Neonatal	N/A	↑ cell size; reorganization of actin filaments into sarcomeric units	[149]

CM, cardiomyocyte; CORT, corticosterone; d, postnatal day; DEX, dexamethasone; GC, glucocorticoid; h, hours; IP, intraperitoneal; LV, left ventricle, m, months; N/A, not applicable; SC, subcutaneous; w, weeks; ↑, increase; ↓, decrease; ↔, no change.

## Data Availability

Not applicable.
